# Perspectives from a GUIDE Practioner

**DOI:** 10.1002/alz70858_110872

**Published:** 2025-12-26

**Authors:** Hillary Lum

**Affiliations:** ^1^ University of Colorado Anschutz Medical Campus, Aurora, CO, USA

## Abstract

As dementia diagnoses rise, the need for comprehensive, person‐centered care becomes more urgent. This part of the session explores the real‐world application of the Guiding and Improved Dementia Experience (GUIDE) Model in dementia care navigation.

